# Complete Mitogenome of “Pumpo” (*Bos taurus*), a Top Bull from a Peruvian Genetic Nucleus, and Its Phylogenetic Analysis

**DOI:** 10.3390/cimb46060320

**Published:** 2024-05-28

**Authors:** Richard Estrada, Deyanira Figueroa, Yolanda Romero, Wuesley Yusmein Alvarez-García, Diorman Rojas, Wigoberto Alvarado, Jorge L. Maicelo, Carlos Quilcate, Carlos I. Arbizu

**Affiliations:** 1 Dirección de Desarrollo Tecnológico Agrario, Instituto Nacional de Innovación Agraria (INIA), Lima 15024, Peru; richard.estrada.bioinfo@gmail.com (R.E.); deyanirafigueroa66@gmail.com (D.F.); yolanda.bioinfo@gmail.com (Y.R.); walvarezg@unc.edu.pe (W.Y.A.-G.); diormanr@gmail.com (D.R.); promegnacional@inia.gob.pe (C.Q.); 2 Facultad de Ingeniería Zootecnista, Agronegocios y Biotecnología, Universidad Nacional Toribio Rodríguez de Mendoza de Amazonas (UNTRM), Cl. Higos Urco 342, Chachapoyas 01001, Peru; wigoberto.alvarado@untrm.edu.pe (W.A.); jmaicelo@untrm.edu.pe (J.L.M.); 3 Facultad de Ingeniería y Ciencias Agrarias, Universidad Nacional Toribio Rodríguez de Mendoza de Amazonas (UNTRM), Cl. Higos Urco 342, Amazonas 01001, Peru

**Keywords:** cattle, NGS, phylogenomics, INIA

## Abstract

The mitochondrial genome of Pumpo (*Bos taurus*), a prominent breed contributing to livestock farming, was sequenced using the Illumina HiSeq 2500 platform. Assembly and annotation of the mitochondrial genome were achieved through a multifaceted approach employing bioinformatics tools such as Trim Galore, SPAdes, and Geseq, followed by meticulous manual inspection. Additionally, analyses covering tRNA secondary structure and codon usage bias were conducted for comprehensive characterization. The 16,341 base pair mitochondrial genome comprises 13 protein-coding genes, 22 tRNA genes, and 2 rRNA genes. Phylogenetic analysis places Pumpo within a clade predominantly composed of European cattle, reflecting its prevalence in Europe. This comprehensive study underscores the importance of mitochondrial genome analysis in understanding cattle evolution and highlights the potential of genetic improvement programs in livestock farming, thus contributing to enhanced livestock practices.

## 1. Introduction

Cattle were domesticated about 12,000 years ago due to their capacity to provide food, transportation, leather, and manure as fertilizer, among others [1,2]. According to FAO [3], the human population is projected to reach 10 billion by 2050, demanding more efficient cattle production. The last census reported that there are 5,156,044 heads of cattle in Peru [4], which is 14.1% higher than what was reported in the 1994 agricultural census. Most of these cattle populations are Creole cattle (64.03%); however, due to the low productivity of these animals, a breeding improvement strategy to take advantage of the heterosis effect is to crossbreed these Creole cattle with other specialized breeds such as Simmental, Brown Swiss, Gyr, and others [5]. This is why in the 1970s, animals of the Simmental breed arrived in Peru, thanks to an agreement with the German Technical Cooperation, whose objective was the use of fresh semen and frozen semen from imported bulls [6].

The Simmental cattle, also known as Fleckvieh, stand out in southern Germany for their ability to reach live weights of up to 850 kg due to their late maturation and intensive fattening [7]. This breed not only provides high yields of milk and meat but is also distinguished by a notable accumulation of proteins, contributing to its popularity in livestock farming due to its excellent fertility and profitability [8,9]. According to the 2022 National Agricultural Survey [10], 238,125 cattle farmers are using high-quality purebred or improved breeders, or employing semen or embryos to reproduce or improve their livestock, including the Simmental breed. Although the exact Simmental cattle population is unknown, there is a growing demand among farmers. In Peru, the first Simmental bull, sent to the National Semen Bank in 2002, initiated the widespread use of artificial insemination for this breed. Offspring produced with semen from bulls of German, Austrian, Canadian, and Swiss origin are distributed nationwide [11]. In 2020, INIA-MINAGRI developed 10 genetic nuclei in various regions, producing 4000 embryos and 710,000 high-quality semen straws to enhance milk and meat production of breeds such as Simmental [12]. In the Amazonas region of Peru, the Simmental breed is crucial for livestock farming, providing sustenance to numerous families due to its hardiness and dual-purpose aptitude, adapting to diverse agroecological zones in provinces like Utcubamba and Bagua, underscoring its importance in the local economy [13].

The study of the mitochondrial genome is pivotal in unraveling genetic diversity and evolutionary history within cattle breeds [14]. Moreover, its application extends to the conservation of indigenous and rare breeds, as demonstrated by studies such as those concerning Zhangmu cattle [15]. Mitochondrial genome sequencing further aids in identifying beneficial genetic variants crucial for genetic improvement programs, as evidenced in the comprehensive assembly of the Simmental cattle genome [16]. Additionally, investigations into the origins and dispersal of *Bos taurus*, including the Simmental breed, benefit from mitochondrial genome analysis, providing valuable insights into phylogenetic structures and adaptive strategies [17].

The objective of this study was to analyze the complete mitogenome of “Pumpo”, a distinguished bull within the Peruvian genetic nucleus of the National Institute of Agrarian Innovation (INIA for its acronym in Spanish), and to perform a phylogenetic analysis. This analysis aims to deepen our understanding of the genetic composition and evolutionary history of the Simmental breed, particularly its adaptation and performance in various agroecological zones, thereby contributing to the improvement of livestock farming practices.

## 2. Materials and Methods

### 2.1. Sampling

The study subject was a Simmental–Fleckvieh breed bull (Peruvian National Register Number 135, born in 2016) from the Central Genetic Nucleus of the National Institute of Agrarian Innovation, located at the Donoso Agricultural Experiment Station (EEA Donoso in Spanish), which is a government herd where a cattle genetic nucleus is established, located in Huaral, Lima (128 masl; 11°31′18″ S and 77°14′06″ W). The bull was healthy and without known genetic diseases. Data registers show that from May 2021 to date, 30,382 semen straws have been collected from Pumpo. Blood sampling was performed at the EEA Donoso and was collected from the bull’s tail using a vacutainer containing EDTA as an anticoagulant and immediately transported to the laboratory for DNA extraction. This study was conducted by following the Peruvian National Law No. 30407: “Animal Protection and Welfare”.

### 2.2. DNA Extraction and Sequencing

Genomic DNA was isolated using the Wizard Genomic DNA Purification Kit (Fitchburg, WI, USA), adhering to the protocols provided by the manufacturer. The integrity and concentration of the isolated genomic DNA were determined using agarose gel electrophoresis and a Qubit 2.0 Fluorometer (ThermoFisher Scientific, Waltham, MA, USA), respectively. Subsequently, an Illumina paired-end (2 × 150 bp) genomic library was prepared according to Illumina’s established procedures (Illumina, San Diego, CA, USA) and sequenced on an Illumina HiSeq 2500 system by GENEWIZ (South Plainfield, NJ, USA). The sequencing library was clustered on a flow cell, which was then placed in the Illumina sequencing instrument as per the manufacturer’s directions. The Illumina Control Software was employed for image analysis and base calling. The raw sequencing data (.bcl files) obtained from the sequencing process were converted into fastq format using the Illumina bcl2fastq 2.17 software, with the protocol allowing for a single mismatch in the index sequence identification.

### 2.3. Assembly and Annotation of the Mitogenome

Adapters and reads of inferior quality were eliminated using the default parameters in the TrimGalore v0.6.7 and Trimmomatic v0.36 software [18]. Utilizing the trimmed data, we assembled the mitochondrial genome via the GetOrganelle [19] pipeline, incorporating tools such as SPAdes v3.11.1 [20], bowtie2 v.2.4.2 [21], and BLAST+ v2.11 [22] in the process. Annotations for the protein-coding genes, transfer RNAs (tRNAs), and rRNA genes within the mitochondrial genome were generated using the automated mitochondrial gene annotators available online through Geseq in the CHLOROBOX web service [23]. This was followed by manual inspection. Analysis of the tRNA secondary structure was conducted using tRNAs-can-SE 2.0 [24]. The termination codon was excluded. Subsequently, 13 protein-coding genes (PCGs) were merged using the Concatenate Sequence Alignment feature, and codon utilization was examined using the relative synonymous codon usage (RSCU) function within MEGA v11 [25]. A graphical representation of the circular mitochondrial genome was produced using OGDRAW v1.3.1 [26].

### 2.4. Phylogenetic Analysis

To ascertain the genetic affiliation of Pumpo, we analyzed 49 mitochondrial genomes from other *Bos* species cataloged in GenBank, complemented by a species from the genus Bison (*Bison bison*), a member of the same subfamily Bovinae, serving as an outgroup (Table S1). Alignment of each genome was conducted using the software MAFFT v7.475 [27], followed by the construction of the most accurate maximum likelihood (ML) tree based on a GTR + GAMMA evolutionary model. This step was succeeded by 1000 nonparametric bootstrap analyses using RAxML v8.2.11 [28]. The inferred phylogenetic trees were visualized using iTOL [29].

## 3. Results

### 3.1. Genome Size and Organization

**Figure 1:**
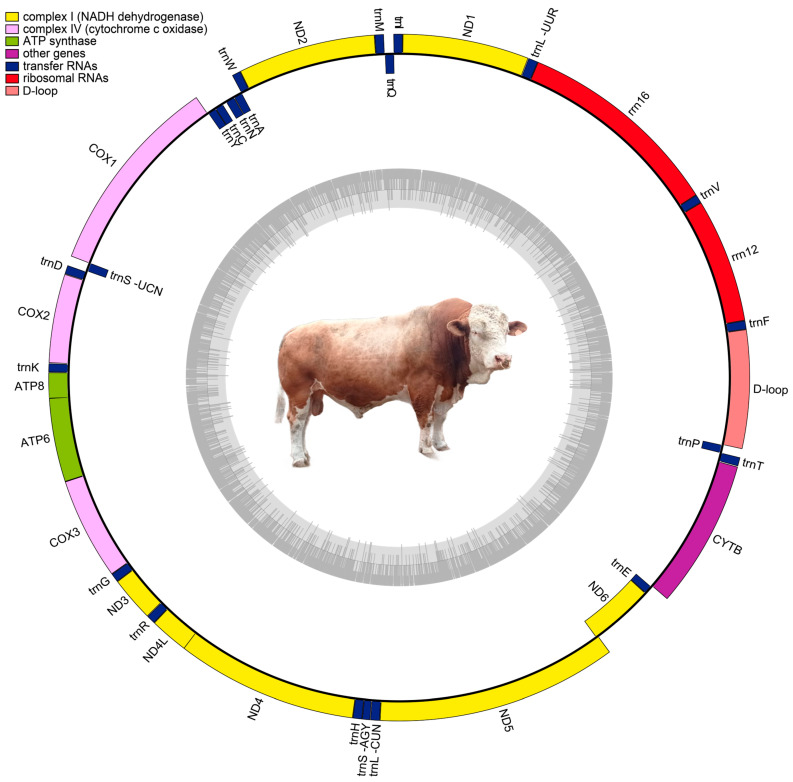
The mitochondrial genome map of Pumpo, a top bull from the Peruvian genetic nucleus of INIA.

The complete mitochondrial genome of Pumpo spans 16,341 base pairs (bp). This genome comprises 13 protein-coding genes, 22 tRNA genes, and 2 rRNA genes (Figure 1). The heavy (H) strand harbored the majority of genes, totaling 27, while the light (L) strand housed 9 genes. The elemental composition of this genome was distributed as follows: 24.44% Adenine (A), 25.13% Thymine (T), 25.62% Cytosine (C), and 24.81% Guanine (G). The most extensive overlap region, spanning 48 bp, is located between the *tRNA*^Leu^ and *Nd5* genes. Additionally, the widest intergenic spacer, covering 32 bp, lies between the *tRNA*^Cys^ and *tRNA*^Tyr^ genes (Table 1). The complete mitochondrial genome sequence has been deposited in the GenBank database under accession number PP780079. The corresponding BioProject, BioSample, and SRA identifiers are PRJNA1097623, SAMN40874274, and SRR28589481, respectively. The assembly coverage was 150×.

### 3.2. Protein Coding Genes (PCGs) and Codon Usage

**Figure 2:**
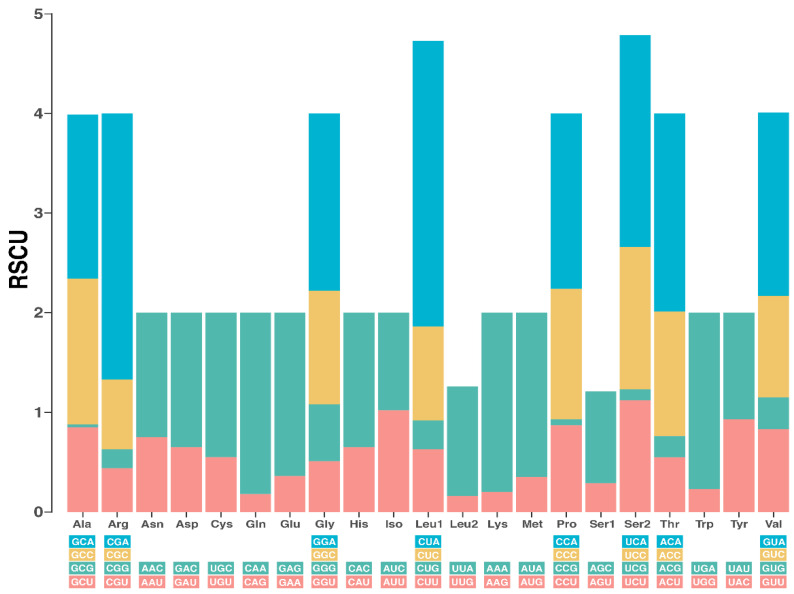
The relative synonymous codon usage (RSCU) of the mitochondrial genome’s protein-coding genes of Pumpo, a top bull from the Peruvian genetic nucleus of INIA.

In the mitogenome of Pumpo, 13 PCGs spanning a total length of 12,309 bp were identified. This accounts for approximately 75.22% of the entire genome. Additionally, this mitochondrial genome is responsible for the synthesis of 4181 amino acids. These PCGs consist of seven NADH dehydrogenase subunits, two ATPase subunits, and a gene corresponding to cytochrome b. It is noteworthy that PCGs exhibit a bias towards AT base composition, ranging from 33.5% for the *Nd4* gene to 95.39% for the *Cox2* gene. Furthermore, the length of PCGs showed wide variability, ranging from 200 bp for *Atp8* to 1772 bp for *Nad5*. In terms of the length of proteins encoded by these genes, it ranged from 66 to 590 amino acids (Table 2). The most frequent start and stop codons were ATG and TAA, respectively. In contrast, the *Nd1*, *Nd2*, *Cox3*, *Nd3*, and *Nd4* genes exhibited incomplete stop codons, represented as TA- or T-. PCGs contained the following five codons with the highest RSCU values: CUA (2.87), CGA (2.67), UCC (2.13), ACA (1.99), and GUA (1.84) (Figure 2).

### 3.3. Ribosomal RNA, Transfer RNA, and Non-Coding Regions

**Figure 3:**
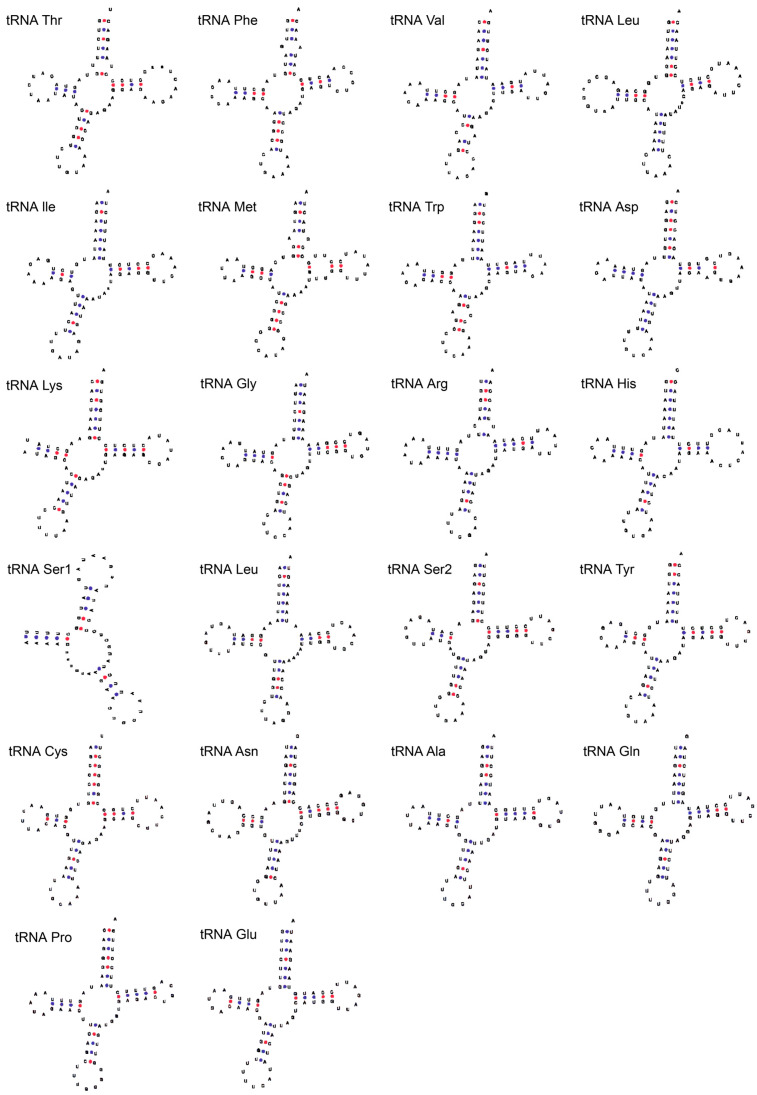
The predicted secondary structures of 22 transfer RNA genes from the mitogenome of Pumpo.

A total of 22 transfer RNA (tRNA) genes were identified, with total lengths ranging from 63 bp for *tRNA*^Phe^ to 74 bp for *tRNA*^Leu2^ (Table 1). The H strand harbored 14 tRNA genes, while the L strand encoded 8 tRNA genes. All these tRNA genes exhibited the characteristic cloverleaf secondary structure, with two exceptions: the *tRNA*^Lys^ and *tRNA*^Ser1^ genes (Figure 3). The total length of the two ribosomal RNA (rRNA) genes (*12S* and *16S*) amounted to 2525 bp. These genes were delimited by *tRNA*^Phe^ and *tRNA*^Leu2^ (Table 1). The control region (*D-loop*), with a total length of 911 bp, was delimited by the *tRNA*^Pro^ and *tRNA*^Phe^ genes (Table 1).

### 3.4. Phylogenetic Analysis

**Figure 4:**
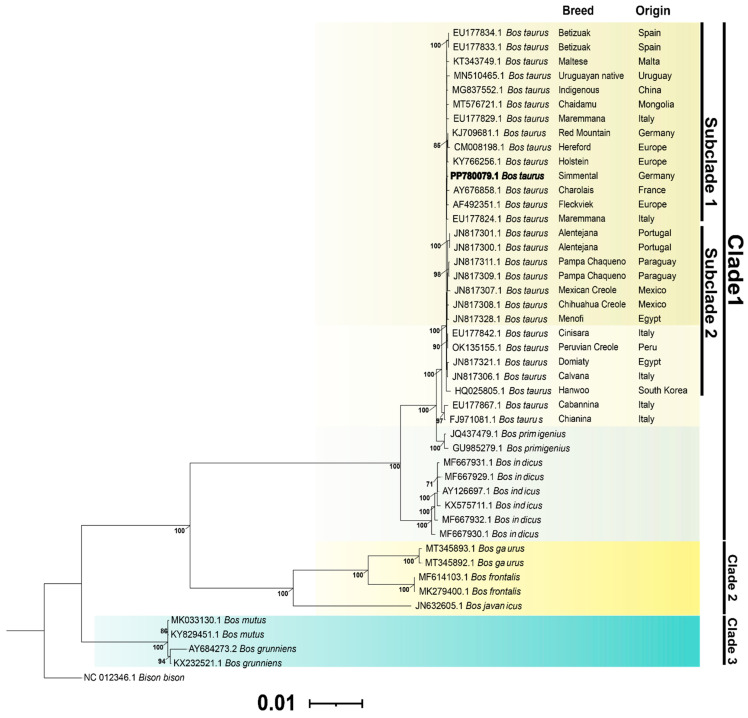
The phylogenetic tree, constructed using maximum likelihood and based on mitochondrial genomic sequences from *Bos* species, displays bootstrap support values exclusively for branches receiving over 70% support. *Bison bison* was designated as the outgroup in this analysis.

An exhaustive phylogenetic analysis of the mitochondrial genomes of *Bos* species available in GenBank was performed using the maximum likelihood (ML) inference methodology, obtaining high bootstrap support values. Three main clades were identified in the phylogenetic tree. The species *B. taurus* was located within a monophyletic clade that is divided into subclade 1 and subclade 2, while *B. primigenius* and *B. indicus* form separate clades. Members of *B. gaurus*, *B. frontalis,* and *B. javanicus* were grouped in clade 2. On the other hand, *B. grunniens* and *B. mutus* constituted clade 3, which is closely related to clades 1 and 2. Pumpo was placed in subclade 1, along with other cattle specimens from France, Germany, Spain, Italy, Uruguay, Mongolia, Malta, and China. On the other hand, subclade 2 was mainly composed of cattle from Italy, Portugal, Mexico, Egypt, Paraguay, and Peru (Figure 4).

## 4. Discussion

Sequencing and characterization of the mitochondrial genome of Pumpo were performed, revealing a length of 16,341 bp. This dimension aligns with sizes observed in other mitogenomes in the Bovinae subfamily, such as *Bos taurus* (16,339 bp), *B. indicus* (16,339 bp), *B. frontalis* (16,340 bp), *B. grunniens* (16,324 bp), and *B. gaurus* (16,345 bp) [30,31,32,33,34]. These findings confirm the similarity in size among mitogenomes in this subfamily. Additionally, the gene order and structure were found to be consistent with those previously reported for *B. taurus* mitogenomes [30,35,36].

The analysis of protein-coding genes in *B. taurus* revealed a pattern of codon usage reflecting the typical bias observed in various organisms such as different *Bos* species [30,35], stemming from a combination of evolutionary and biological factors [37]. The convergence of these codon usage patterns suggests the influence of lineage-specific factors, including translational selection, tRNA availability, and protein structure, as highlighted in previous studies [38,39]. Understanding how these patterns affect protein structure and function can shed light on the biology and evolution of domestic cattle, as well as have practical applications in genetic improvement and agricultural biotechnology [40].

To comprehend the evolutionary connection of Pumpo, a phylogenetic tree was constructed using maximum likelihood inference methodology alongside other cattle breeds and *Bos* species. The mitochondrial genome phylogeny exhibited similarities with those of other *Bos* genera [30,31,33]. The phylogenetic tree displayed three taxonomic clusters, with the first clade experiencing a bifurcation resulting in the formation of two distinct subclades. Pumpo is grouped within subclade 1, where European cattle predominate, given that this Simmental breed is particularly prevalent in Europe [41]. This breed has been extensively researched; a comprehensive analysis of genetic variability revealed complex phylogenetic patterns, reflecting diverse genetic and selective influences over time [42]. Furthermore, the study of identifying candidate genes associated with key productive and reproductive traits underscores the significance of human selection in the genetic evolution of this breed [43]. Subdivision of the *B. taurus* and *B. indicus* species was also observed [44,45]. Pumpo was part of a genetic group that has been the subject of a population genetics study using SNP data. This analysis revealed a significant correlation with specimens of the Simmental breed [46].

Mitogenomes are crucial in evolutionary studies and forensic applications, presenting significant advantages and limitations. The precise assembly of mitogenomes is challenging due to errors and missing sequences from short-read sequencing, although long-read strategies improve accuracy [47]. Additionally, certain mitogenes are more prone to transfer between mitochondria and the nucleus, causing losses or parallel transfers in different lineages [48]. Despite limitations, mitogenomes provide valuable information about the evolutionary history and genetic diversity of *Bos* cattle, including evidence of genetic contributions from ancient Chinese cattle to southern Chinese taurine cattle [49]. To enhance research, it is crucial to explore emerging technologies such as long-read sequencing and integration with nuclear genomic data, which will contribute to a better understanding of evolutionary dynamics and conservation of cattle species, including Pumpo.

## 5. Conclusions

The sequencing and analysis of the mitochondrial genome of Pumpo confirmed its similarity in size and structure to other mitogenomes within the Bovinae subfamily, particularly *B. taurus*. The examination of protein-coding genes in *B. taurus* revealed codon usage patterns typical of various organisms, shedding light on evolutionary and biological influences. Furthermore, phylogenetic analysis placed Pumpo within a clade predominantly occupied by European cattle breeds, indicating its evolutionary relationship with these lineages. Despite challenges in mitogenome assembly and gene transfer, mitogenomes remain invaluable for understanding the evolutionary history and genetic diversity of *Bos* cattle. Continued exploration of advanced sequencing technologies and integration with nuclear genomic data will further enhance our understanding of Pumpo and other cattle species’ evolutionary dynamics and conservation needs.

## Figures and Tables

**Table 1:** Gene organization of the mitochondrial genome of Pumpo cattle.

Gene	Nucleotide Positions	Size (bp)	Strand *	Intergenic Spacer (bp)
*tRNA* ^Phe^	365–432	68	H	
*12S rRNA*	433–1388	956	H	
*tRNA* ^Val^	1389–1455	67	H	
*16S rRNA*	1456–3026	1571	H	
*tRNA* ^Leu2^	3027–3101	75	H	
*Nd1*	3104–4059	956	H	2
*tRNA* ^IIe^	4060–4128	69	H	
*tRNA* ^Gin^	4126–4197	72	L	−1
*tRNA* ^Met^	4200–4268	69	H	2
*Nd2*	4269–5310	1042	H	
*tRNA* ^Trp^	5311–5377	67	H	
*tRNA* ^Ala^	5379–5447	69	L	
*tRNA* ^Asn^	5449–5521	73	L	1
*tRNA* ^Cys^	5554–5620	67	L	2
*tRNA* ^Tyr^	5621–5688	68	L	
*Cox1*	5690–7234	1545	H	1
*tRNA* ^Ser2^	7332–7302	31	L	−1
*tRNA* ^Asp^	7307–7378	72	H	4
*Cox2*	7377–8060	684	H	−1
*tRNA* ^Lys^	8064–8130	67	H	3
*Atp8*	8132–8332	201	H	1
*Atp6*	8293–8973	681	H	60
*Cox3*	8973–9756	784	H	−1
*tRNA* ^Gly^	9757–9825	69	H	
*Nd3*	9826–10,171	346	H	
*tRNA* ^Arg^	10,173–10,241	69	H	−1
*Nd4L*	10,242–10,538	297	H	
*Nd4*	10,532–11,909	1378	H	−5
*tRNA* ^His^	11,910–11,979	70	H	
*tRNA* ^Ser^	11,980–12,039	60	H	
*tRNA* ^Leu^	12,041–12,111	71	H	1
*Nd5*	12,112–13,932	1821	H	
*Nd6*	13,916–14,443	528	L	−17
*tRNA* ^Glu^	14,444–14,512	69	L	
*CytB*	14,514–15,656	1143	H	1
*tRNA* ^Thr^	15,661–15,729	69	H	4
*tRNA* ^Pro^	15,729–15,794	66	L	−1
*D-loop*	1–364, 15,795–16,341	364, 547	H	

* Strand: H (Heavy), L (Light).

**Table 2:** Features of protein-coding genes detected in the mitochondrial genome of Pumpo cattle.

Gene	Gene Length (bp)	A + T Content (%)	Start/Stop Codon	Protein Length (aa)
*Nd1*	955	85.79	ATG/TA-	319
*Nd2*	1041	86.2	ATG/TA-	348
*Cox1*	1544	80.3	ATG/TAA	514
*Cox2*	750	95.39	ATG/TAA	227
*Atp8*	200	57	ATG/TAA	66
*Atp6*	679	50	ATG/TAA	227
*Cox3*	783	57.25	ATG/TA-	262
*Nd3*	346	57.54	ATA/TA-	116
*Nd4L*	296	52.56	ATG/TAA	98
*Nd4*	1377	33.5	ATG/T-	460
*Nd5*	1772	77.32	ATA/TAA	590
*Nd6*	527	59.02	ATG/TAA	175
*CytB*	1139	54.86	ATG/AGA	379
Total	12,309			4181

## Data Availability

The associated Bioproject, Biosample, and Sequence Read Archive (SRA) numbers are PRJNA1097623, SAMN40874274, and SRR28589481, respectively.

## Supplementary Materials

The following supporting information can be downloaded at: https://www.mdpi.com/article/10.3390/cimb46060320/s1, Table S1. Species, breed, origin, accession code, and clade assignment of the 45 individuals examined in this study.
